# Presence of purpura is related to active inflammation in association with IL-5 in eosinophilic granulomatosis with polyangiitis

**DOI:** 10.1007/s00296-020-04672-8

**Published:** 2020-08-07

**Authors:** Hiroshi Kataoka, Tomoko Tomita, Makoto Kondo, Masaya Mukai

**Affiliations:** Department of Rheumatology and Clinical Immunology, Sapporo City General Hospital, Sapporo, Japan

**Keywords:** Antineutrophil cytoplasmic antibody, Churg–Strauss syndrome, Eosinophils, Interleukin-5, Purpura

## Abstract

Eosinophilic granulomatosis with polyangiitis (EGPA) is a relatively rare necrotizing vasculitis that causes asthma, nasal involvement, peripheral nerve disturbance, renal disorder, and cutaneous lesions like purpura and is characterized by eosinophil infiltration into the damaged tissue. Purpura is the most common cutaneous lesion, but it remains unknown whether this skin lesion is associated with disease activity of EGPA and laboratory data including interleukin (IL)-5, a target cytokine of this disease. We conducted a search of our hospital electronic records for cases of EGPA from the last 10 years. Symptoms related to EGPA (fever, asthma, nasal and cutaneous manifestations, neuropathy), the Birmingham Vasculitis Activity Score (BVAS), and laboratory parameters, such as eosinophil count, urinalysis, antineutrophil cytoplasmic antibody (ANCA), CRP, IgE and IL-5, before and during treatment were compared among the eligible cases. A total of 28 EGPA patients (21 females and 7 males) were selected. Almost all developed peripheral neuropathy. Fever occurred in 25%, nasal symptoms in 38.1% and purpura in 44%. Glomerulonephritis developed in 7.7%. One patient had cardiac involvement (3.6%). The laboratory data showed a marked increase in peripheral eosinophil count, CRP, serum IgE and serum IL-5. ANCA was positive in 15.4%. In the univariate analysis, presence of purpura was associated with increased CRP and IL-5, and high BVAS score. Multivariate analysis revealed a robust relationship between purpura and CRP. Our findings showed that presence of purpura was associated with increased CRP and IL-5, and high disease activity in EGPA.

## Introduction

Eosinophilic granulomatosis with polyangiitis (EGPA) is a relatively rare necrotizing vasculitis resembling polyarteritis nodosa, which causes asthma, nasal involvement, peripheral nerve disturbance, heart and renal disorders, lymphadenopathy, cutaneous lesions like purpura and subcutaneous nodules with eosinophil infiltration into perivascular area and prominent eosinophilia [1, 2]. The annual incidence of EGPA in Japan is 2.4 per million a year [3]. Prognosis is relatively poor, as 30–40% of patients experience relapse and mortality is 5–10% [4, 5]. In terms of classification of vasculitis, EGPA is a less common type of antineutrophil cytoplasmic antibody (ANCA)-related vasculitis, and 30–50% of EGPA cases are positive for ANCA [6]. EGPA in the presence of heart and gastrointestinal lesions, and proteinuria, showed higher relapse rate [5]. Corticosteroid is essentially used for the treatment of this disease, and according to the severity of visceral damage, cyclophosphamide, azathioprine and biologic agents are concomitantly given to improve cardiac, gastrointestinal and neurological involvement [7, 8]. To prevent irreversible damage in organs and nerves, treatment for EGPA should be commenced as soon as possible. However, there is a delay of 5–24 days from the onset because no reliable biomarker currently exists and the diagnosis of EGPA needs to be made on the basis of general consideration of clinical course and the laboratory data [8–10].

Skin involvement occurs in two-thirds of patients with EGPA, and when located at the area of the peripheral neuropathy, it is one of the clinical clues to diagnose EGPA [5, 11]. In this study, we sought to determine whether the presence of cutaneous involvement was associated with diagnosis of EGPA and decision of treatment by retrospectively analyzing manifestations, laboratory data and the factors that affected disease activity and treatment.

## Materials and methods

### Patients

The study design was a retrospective cross-sectional study in a single rheumatology center. Patients of EGPA were selected by searching electronic records using keywords of the disease names, such as eosinophilic granulomatosis with polyangiitis (EGPA), allergic granulomatous angiitis (AGA) and Churg–Strauss syndrome (CSS) for the recent 10 years (2009–2018). Enrolled patients were 18 years or older and their condition had been diagnosed as EGPA, AGA, or CSS and received induction and maintenance therapy for 6 months or more at our hospital. Patients who lacked detailed information at diagnosis or had been treated during the induction phase at other hospitals and whose subsequent maintenance therapy was taken over at our hospital were excluded. Diagnosis of EGPA was reconfirmed according to the American College of Rheumatology 1990 Criteria [12] on the basis of their history of the disease, results of physical examination, blood tests, imaging, nerve conduction test, and other information that were required for the diagnosis. Peripheral neuropathy was clinically diagnosed by neurologists at our hospital based on the nerve conduction test. Cutaneous involvement was diagnosed by dermatologists at our hospital primarily on the basis of clinical findings and the pathology of skin lesions with the patients’ consent. Nasal lesions were diagnosed by otorhinolaryngologists at our hospital with reference to imaging studies and pathology. Systemic vasculitis activity was evaluated using the Birmingham Vasculitis Activity Score (BVAS) version 3 [13]. Symptoms at the onset of EGPA, changes in the laboratory parameters (eosinophil count, C-reactive protein [CRP], immunoglobulin [Ig] E, myeloperoxidase [MPO]-ANCA and interleukin [IL]-5) during treatment, initial and recent dose of prednisolone, and history of additional use of immunosuppressants were compared among the eligible cases. Quantification of IL-5 in the stored sera from the patients was performed using enzyme immunoassay at BML (Bio Medical Laboratories) Inc. The cut-off level that the company set was 4 pg/ml.

### Bioethics

This was a retrospective observational study, and the protocol was approved by the ethical committee of our hospital (H30-059-515); all participants provided written informed consent in accordance with the Declaration of Helsinki. Patients were notified of the protocol of this study at our hospital and could opt out of the patient list at any time.

### Statistical analysis

Concerning statistical analysis, univariate analysis by unpaired *t* test or the Mann–Whitney *U* test was performed for comparison of each characteristic factor between patients with or without cutaneous lesions, after assessing the normality of the data using the D’Agostino and Pearson test as indicated in each table with the Prism 8 (version 8.4.1, GraphPad Software, LLC). The parameters with significant difference in the univariate analysis, were used in the multivariate linear regression analysis. In every analysis, a two-tailed *p* value less than 0.05 was considered as statistically significant.

## Results

### Symptoms and laboratory data

**Fig. 1:**
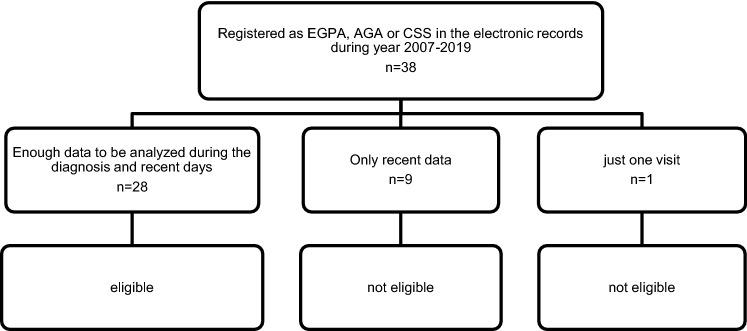
Patient disposition. *EGPA* eosinophilic granulomatosis with polyangiitis, *AGA* allergic granulomatous angiitis, *CSS* Churg-Strauss syndrome

**Fig. 2:**
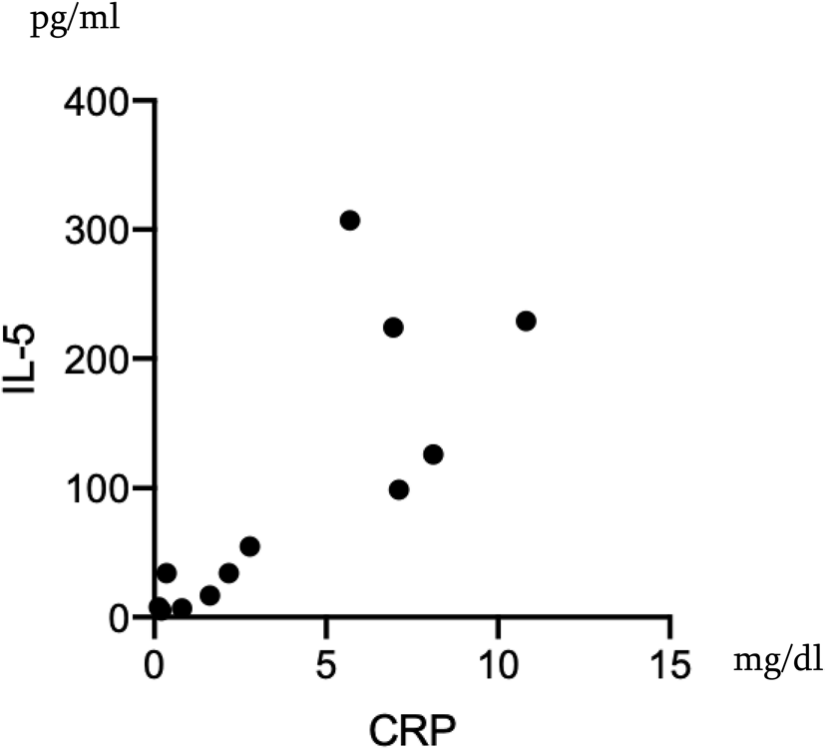
Correlation of Serum IL-5 and CRP at the Onset of EGPA. Each plot represents each patient. Spearman *r*^2^ = 0.63 (*p* = 0.0006)

**Fig. 3:**
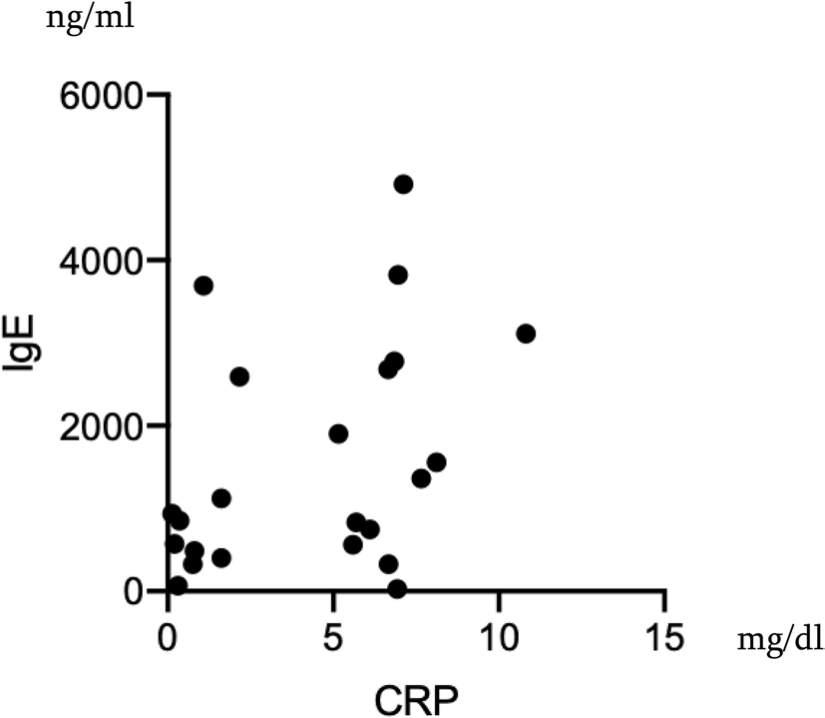
Correlation of Serum IgE and CRP at the Onset of EGPA. Each plot represents each patient. Spearman *r*^2^ = 0.16 (*p* = 0.038)

A total of 38 patients (28 females and 10 males) were selected as EGPA from our records (Fig. 1). Of 38 patients, 28 (21 females and 7 males) had well-documented medical history about symptoms, laboratory data, images, pathology and medication that were eligible for further analysis (Table 1). Of 38 EGPA patients, nine patients had only recent records and data during quiescent state of the disease, and one patient visited our clinic just once. Thus, these ten patients were excluded from this study. The average age of the eligible 28 patients was 56 years and average disease duration was 4.5 years (Table 1). Of various initial symptoms, almost all patients (96.2%) developed peripheral neuropathy. Fever occurred in 25%, nasal symptom in 38.1% and cutaneous lesions in 44%. Of 28, 3 patients whose records lacked description about skin involvement were excluded from further analysis. Body weight loss of 2 kg or more per month occurred in 30%. Urinalysis revealed that 7.7% (2 patients) showed hematuria and proteinuria. The renal biopsy revealed crescentic glomerulonephritis. One patient had cardiac involvement (3.6%). In their laboratory data, there was a marked increase in peripheral eosinophil count (10,639 ± 9615 /μL), CRP (4.2 ± 3.2 mg/dl), serum IgE (1514 ± 1358 ng/ml) and serum IL-5 (94.1 ± 101.1 pg/ml). Including the two patients with nephritis as above, 15.4% (4 patients) of the patients showed positive ANCA (all MPO-ANCA, 179.3 ± 98.4 IU/ml). Initial CRP level correlated with serum levels of IL-5 and IgE (*r*^2^ = 0.63 [*p* = 0.0006], 0.16 [*p* = 0.038], respectively) (Figs. 2, 3). The median BVAS score was 10 (range 2–23). Presence of cutaneous lesions was associated with elevated CRP, serum IL-5 and high BVAS score (Tables 2, 3). Multivariate linear regression analysis of CRP and IL-5 showed that only CRP was related to the presence of purpura (*p* = 0.0223, 95% confidence interval 0.022–0.21, Table 3). In contrast, cutaneous lesions were irrespective of presence of nasal lesions, neurological disturbance and renal involvement or eosinophil count or serum IgE level (Table 2). Eosinophil count and CRP were not related to the BVAS score (data not shown). All cutaneous lesions were purpura, and skin biopsy was performed on 11 of them. The pathological findings were typical leukocytoclastic vasculitis in eight patients (73%), and massive eosinophilic infiltration with other inflammatory cells in dermis was found in three patients. Concerning allergens, specific IgE was tested in seven patients. The allergens were Japanese white birch in one patient and candida in another, whereas no specific allergen was detected in five of them.

**Table 1:** Demographic data of EGPA patients

EGPA	*n* = 28
Age (year)	56 ± 15
Disease duration (year)	4.5 ± 2.5
Fever (%)	25.0
Weight loss over 2 kg/month (%)	30.0
Nasal/sinus lesions (%)	38.1
Cutaneous lesions (%)	44.0
Peripheral neuropathy (%)	96.2
Renal lesions (%)	7.7
BVAS score (median, range)	10 (2–23)

*BVAS score* Birmingham Vasculitis Assessment Score ver. 3.0

**Table 2:** Symptoms at the Onset of EGPA with or without Cutaneous Lesions

Cutaneous lesions (*n* = 25)	+	−	*p*
Patients	11	14	**−**
Age (year)	58.7	54.5	n.s
Fever (%)	30	16.7	n.s
Nasal/sinus lesions (%)	55.6	27.3	n.s
Cutaneous lesions (%)	100	0	< 0.01*
Peripheral neuropathy(%)	100	92.9	n.s
Renal lesions (%)	10	7.1	n.s
BVAS score	14 ± 4	9 ± 4	< 0.01**

Statistical analysis was done by *Fisher’s exact test, **Mann–Whitney test

**Table 3:** Parameters of EGPA at the onset with or without cutaneous lesions

	Cutaneous lesions (*n* = 25)		Univariate	Multivariate	
	+	−	*p*	*p*	95% CI
Eosinophils (/μL)	7648 ± 4699	13,635 ± 12,135	0.26	–	–
CRP (mg/dl)	6.2 ± 2.7	3.2 ± 2.8	0.022^*^	0.0223	0.022–0.21
Positive ANCA (%)	20	8.3	0.99	–	–
IgE (IU/ml)	1728 ± 1447	1233 ± 1153	0.52	–	–
IL-5 (pg/ml)	155.8 ± 103.1	50 ± 78.8	0.0303^**^	0.93	− 0.0034–0.0032

Statistical Univariate analysis was done by *t test, **Mann–Whitney test

### Treatment and follow-up

Methylprednisolone pulse therapy was introduced to 61.5% of the patients (Table 4). Initial prednisolone (PSL) was 53.6 ± 10.4 mg/day. Concomitant therapy included intravenous cyclophosphamide (IVCY) (16%), intravenous immunoglobulin (IVIG) (34.6%), rituximab (RTX) (7.1%), azathioprine (AZP) (19.2%), inhalant corticosteroid (ICS) (26.9%) and pregabalin (42.3%). In their treatment planning, cutaneous lesions did not affect the doctor’s decision on mPSL pulse, initial PSL dose, IVCY, IVIG, RTX, AZP, ICS and pregabalin (Table 4). RTX was introduced to two patients with crescentic glomerulonephritis.

**Table 4:** Comparison of treatment for EGPA between patients with and without cutaneous lesions

Cutaneous lesions (*n* = 25)	+	−	*p*
mPSL pulse (%)	81.8	50	0.208^a^
Initial PSL dose (mg/dl)	56.4 ± 8.1	51.5 ± 12.1	0.2739^b^
IVCY (%)	30	7.1	0.2721^a^
IVIG (%)	30	42.9	0.6785^a^
AZP (%)	10	21.4	0.6146^a^
ICS (%)	27.3	21.4	> 0.99^a^
Pregabalin (%)	50	28.6	0.4028^a^
Rituximab (*n*)	1	1	–

*mPSL* methylprednisolone, *PSL* prednisolone, *IVCY* intravenous cyclophosphamide injection therapy, *IVIG* intravenous immunoglobulin, *AZP* azathioprine, *ICS* inhalant corticosteroid

Statistical analysis was done by ^a^Fisher's exact test, ^b^*t* test

Total follow-up observation time was 3.7 ± 2.3 years. All enrolled patients visited our clinic every 4–12 weeks. The average eosinophil count was 255 /μL and mean CRP was 0.32 at the recent visit. PSL dose was 5.6 ± 2.4 mg/day at the final visit during the observation period. One case was omitted from calculating the observation time and PSL dose because she continued treatment at an adjacent hospital after our care and recent dose was unknown. One patient required increases in PSL dose (from 5 to 40 mg/day) for the development of interstitial pneumonia (IP) in spite of remission for over a year. The patient recovered from IP and PSL was reduced to 5 mg/day at the recent visit. No other patients experienced recurrence that required addition of PSL or immunosuppressant. There was no flare of glomerulonephritis in two patients and they did not require re-treatment with RTX. Mepolizumab was introduced to three patients at 1.2 years (median) from the onset because of recurrence of eosinophilia in all cases and worsening of asthma even after using constant ICS inhalation in one case. Follow-up IL-5 data were available in four patients including two patients using mepolizumab. IL-5 was tested in the most recently stored sera (those obtained before adding mepolizumab in cases treated with the biologic agent), and the results were under the cut-off level (4 pg/ml) in all patients.

## Discussion

In this study, the presence of purpura was significantly associated with CRP, IL-5 and high BVAS score, suggesting that cutaneous lesions reflected high disease activity of EGPA (Tables 2, 3). Multivariate analysis showed that CRP was the most related marker to purpura (Table 3).

Cutaneous lesions as clinical manifestations in EGPA include purpura, which occurs in 25% of patients and is frequently found in their legs, nodules, urticaria, livedo and ulcers [14]. A French cohort of 383 patients also showed that, of the 39.7% of patients with skin lesions, purpura occurred in 22.5%, urticaria in 9.9%, subcutaneous nodules in 9.7%, livedo reticularis in 3.9% and gangrenous necrotic lesions in 3.7% [4]. In our patients, purpura mainly developed in all patients with cutaneous involvement, and urticaria or livedo-like lesions were partly found in some cases. Leukocytoclastic vasculitis was the major findings in skin pathology, and 27% showed massive eosinophil infiltration indicating eosinophilic inflammation in EGPA.

IL-5 is a cytokine that belongs to the *β* common chain family and mediates the differentiation and migration of eosinophil from blood to target tissue [15]. Th2 cells, mast cells, CD34^+^ progenitor cells, invariant natural killer T cells, group 2 innate lymphoid cells and eosinophils are major cellular sources of IL-5 [15, 16]. This cytokine stimulates eosinophil precursors to synthesize granule proteins, such as major basic protein (MBP), eosinophil-derived neurotoxin (EDN), eosinophil peroxidase (EPX) and eosinophil cationic protein (ECP) [17]. IL-5 inhibits eosinophil adhesion to bone marrow sinus endothelium, resulting in mobilization of eosinophils to the peripheral tissues in conjunction with eotaxin. At the inflammation site, the recruited eosinophils are activated and degranulated to release the enzymes that cause tissue damage. Finally, dying eosinophils release self-DNA that forms extracellular DNA nets, which accelerates eosinophilic inflammation [17, 18]. As a result, systemic vasculitis in the skin, kidney, nerve, nasal cavity, and lung occurs along with the increased CRP in EGPA. Therefore, high CRP in patients with cutaneous lesions was thought to be a result of active eosinophilic inflammation of EGPA in relation to elevated IL-5.

Eosinophils in the peripheral blood produce massive IL-25 in EGPA [19]. IL-25 is also released from damaged epithelial cells, along with IL-33, as an alarmin [6]. These two cytokines stimulate type 2 innate lymphoid cells to induce IL-5 [6, 20]. Activated antigen-presenting cells, such as DC and IL-33, activate IL-33 receptor-expressing Th2 cells (i.e. pathogenic Th2 cells) to produce IL-5 [21, 22]. In this way, eosinophils promote and enhance Th2 response in the inflammatory site.

This study has certain limitations. First, the number of the eligible patients was small and second, this was a retrospective study conducted in a single center. Treatment strategy of each patient was dependent on a patient’s choice or a doctor’s decision. The results of this study do not offer the best choice, such as PSL monotherapy or the addition of IVCY or mepolizumab, for each case based on the presence or absence of purpura, although the presence of purpura indicated the disease activity of EGPA. A multicenter prospective cohort study may reveal the requirement of immunosuppressants or biologics for EGPA with purpura as the diagnosis. Moreover, as mepolizumab inhibits IL-5 directly, the efficacy of this biologic agent would elucidate how IL-5 is involved in the development of cutaneous lesions.

We have concluded that a cutaneous lesion like purpura is an important index indicating robust inflammation in connection with increased IL-5 and eventually high disease activity of EGPA. Further study with more patients enrolled may provide better choices of combination of PSL, IVCY, IVIG, and anti-IL-5 therapy on the basis of clinical and laboratory findings.

## Data Availability

The datasets concerning the current study made on Microsoft Word for Mac version 16.37 and Microsoft Excel for Mac version 16.37 are available from the corresponding author on reasonable request.
